# Efficacy and safety of tranexamic acid administration for subarachnoid hemorrhage: a systematic review and meta-analysis

**DOI:** 10.3389/fneur.2025.1617817

**Published:** 2025-06-17

**Authors:** Eriya Imai, Hiroshi Ito, Hiromu Okano, Akihiko Inoue, Takero Terayama, Hiroshi Okamoto, Toru Hifumi, Yoshihisa Fujimoto, Gaku Fujiwara, Yasuhiro Kuroda

**Affiliations:** 1 Division of Anesthesia, Mitsui Memorial Hospital, Chiyoda-ku, Tokyo, Japan; 2 Department of Traumatology and Acute Critical Medicine, Osaka University Graduate School of Medicine, Osaka, Japan; 3 Department of Critical Care Medicine, St. Luke's International Hospital, Chuo-ku, Tokyo, Japan; 4 Department of Emergency and Critical Care Medicine, Hyogo Emergency Medical Center, Chuo-ku, Kobe, Japan; 5 Department of Emergency, Self Defense Forces Central Hospital, Setagaya-Ku, Tokyo, Japan; 6 Department of Emergency and Critical Care Medicine, St. Luke’s International Hospital, Chuo-ku, Tokyo, Japan; 7 Department of Emergency and Critical Care Medicine, St. Marianna University School of Medicine Hospital, Miyamae-ku, Kawasaki, Japan; 8 Department of Management of Technology and Intellectual Property, School of Public Health, Kyoto University, Kyoto, Japan; 9 Department of Emergency, Disaster, and Critical Care Medicine, Faculty of Medicine, Kagawa University, Miki, Japan

**Keywords:** meta-analysis, subarachnoid hemorrhage, systematic review, tranexamic acid, rebleeding

## Abstract

**Introduction:**

Aneurysmal subarachnoid hemorrhage (SAH) carries a high risk of early rebleeding and worsens prognosis. Tranexamic acid (TXA), an antifibrinolytic agent, can prevent rebleeding; however, its effects on mortality and neurological outcomes remain controversial.

**Methods:**

This review evaluated the efficacy and safety of TXA with SAH. MEDLINE, CENTRAL, EMBASE, ICTRP, and ClinicalTrials.gov were systematically searched for randomized controlled trials (RCTs) and non-randomized studies of interventions (NRSIs) to assess TXA use in SAH. Studies comparing TXA with controls with SAH were included. The primary outcome was the mortality; secondary outcomes included neurological outcomes, rebleeding, thromboembolism, delayed cerebral ischemia (DCI), hydrocephalus, and adverse events. The certainty of evidence was evaluated using the Grading of Recommendations, Assessment, Development, and Evaluations (GRADE) approach.

**Results:**

Fifteen RCTs (3,109 patients) and nine NRSIs (1,506 patients) were included. RCTs demonstrated that TXA likely does not reduce mortality (risk ratio [RR], 1.00; 95% confidence interval [CI], 0.82–1.22; moderate certainty) and neurological outcome, and may not increase thromboembolism and DCI. However, TXA probably reduces rebleeding but probably increases hydrocephalus. The NRSIs results were similar.

**Discussion:**

Although routine use is not supported, TXA may be considered for high-risk patients when early aneurysm treatment is unavailable.

**Systematic review registration:**

https://osf.io/yp78b/.

## 1 Introduction

Aneurysmal subarachnoid hemorrhage (SAH) accounts for 5% of all stroke incidents and has a poor prognosis (1). Rebleeding, which occurs most frequently within the first 24 h after the initial hemorrhage, markedly worsens prognosis (2). Although early aneurysm treatment is recommended to prevent rebleeding (2), immediate surgical or endovascular intervention is not always feasible for all patients (2). Tranexamic acid (TXA), an antifibrinolytic agent, has been considered a potential alternative to reduce the risk of rebleeding in such cases. Several studies have reported that TXA effectively decreases the incidence of rebleeding after SAH (3, 4).

Although TXA reduces re-bleeding, its long-term neurological effects remain unclear. Recent randomized controlled trials (RCTs) found no significant effect on neurological outcomes or mortality (5), leading to guideline recommendations against routine use (6). Nevertheless, TXA is still administered in certain settings due to uncertainties regarding the optimal duration (e.g., 24 vs. 72 h) and associated risks (7–9). Previous systematic reviews and meta-analyses (SR/MAs) often combined RCTs and observational studies without distinguishing study designs (9–11), contributing to clinical ambiguity.

This SR/MA evaluated the benefits and risks of TXA in SAH management by analyzing both RCTs and observational studies. Subgroup analyses based on treatment duration and application of the Grading of Recommendations, Assessment, Development, and Evaluation (GRADE) framework assessed the impact of TXA on rebleeding, neurological outcomes, and complications such as thromboembolism and delayed cerebral ischemia (DCI).

## 2 Methods

### 2.1 Protocol and registration

We followed the Preferred Reporting Items for Systematic Review and Meta-Analysis (Supplementary Table S1) (12). This study protocol has been made public under the Open Science Framework (accessible online: https://osf.io/yp78b/ [accessed on July 8, 2024]).

### 2.2 Eligibility criteria

RCTs and non-randomized studies of interventions (NRSI) on TXA for SAH were included regardless of publication status, language, country, observation period, or publication year. Studies on interventions other than TXA, comparisons of TXA with other clotting agents, and studies on intracranial hemorrhage other than spontaneous SAH were excluded. In addition, case reports and case series were excluded.

### 2.3 Participant types

Patients with symptoms of cerebral aneurysmal SAH confirmed by computed tomography (CT), magnetic resonance imaging (MRI), angiography, or cerebrospinal fluid (CSF) analysis and presenting within 72 h of symptom onset were included. Eligible patients were adults aged ≥ 18 years regardless of sex or racial background. Patients with intracranial hemorrhage due to trauma, arteriovenous malformations, or traumatic SAH were excluded.

#### 2.3.1 Intervention type

TXA was administered orally or intravenously. We used a placebo, such as saline, or standard therapy alone as a control.

#### 2.3.2 Outcome types

The following primary and secondary outcomes were evaluated.

##### 2.3.2.1 Primary outcome

The primary outcome was all-cause mortality, including death from re-bleeding, cerebral ischemia, hydrocephalus, extracranial causes, and surgery- or anesthesia-related complications. Follow-up lasted for at least 3 months from the onset of cerebral aneurysmal SAH.

##### 2.3.2.2 Secondary outcomes

The secondary outcomes included neurological outcomes, rebleeding, DCI, hydrocephalus, and adverse events.

Good neurological outcomes were defined as a favorable functional status based on the modified Rankin Scale (mRS) or Glasgow Outcome Scale (GOS), with mRS scores of 0–2 and GOS scores of 4–5 considered indicative of good outcomes (13). Follow-up lasted for at least 3 months from the onset of cerebral aneurysmal SAH.

Rebleeding was defined as bleeding confirmed by CT, autopsy, CSF analysis, sudden changes in vital signs, or neurological deterioration suggestive of rebleeding. The observation period included in-hospital events or those occurring within 24 h of SAH onset.

Thromboembolism includes any form of thrombosis, including clinically suspected or diagnosed deep vein thrombosis of the lower extremities and pulmonary embolism. Follow-up was conducted for a minimum of 3 months after the onset of SAH.

DCI was defined as cerebral ischemia or infarction identified through clinical assessment, CT or MRI, cerebral angiography, or relevant laboratory studies. The observation period included events that occurred during hospitalization.

Hydrocephalus was defined as a gradual onset of disorientation, CT-confirmed ventricular enlargement, and no alternative explanation for deterioration. Follow-up was conducted for a minimum of 3 months after the onset of SAH.

Adverse events were defined according to the criteria set by the original authors. The incidence proportion was calculated as the number of patients experiencing any adverse event, excluding events without specific definitions, divided by the total number of patients.

For all secondary outcomes, definitions provided by the original authors were accepted.

### 2.4 Search strategy and study selection

We searched MEDLINE (PubMed) from inception through May 30, 2024; the Cochrane Central Register of Controlled Trials (Cochrane Library); EMBASE (Dialog) from inception through May 15, 2024; and ongoing or unpublished trials, including the World Health Organization International Clinical Trials Platform Search Portal (WHO ICTRP) and ClinicalTrials.gov from inception through May 15, 2024 (Supplementary Table S2). The original authors were also asked for unpublished or additional data. Furthermore, the reference lists of eligible studies, relevant articles, and international guidelines (6) were reviewed.

Two reviewers (EI and HI) independently screened the titles and abstracts of all the identified studies. Articles selected during the abstract screening underwent a full-text review for eligibility. If needed, the original authors were contacted to resolve content-related discrepancies. Disagreements were resolved by consensus or by a third reviewer (HO).

### 2.5 Data extraction

Two independent reviewers (EI and HI) extracted the data from the included trials using a standardized data collection form. The collected data included the author, publication year, study design, setting, sample size, sex, age, eligibility criteria, country, TXA dosage, administration route, treatment duration, ischemia prevention methods, neurological status at admission, post-treatment course, and outcomes. Trials with missing data were requested from the study authors, and trials with unretrievable data were excluded.

### 2.6 Risk of bias assessment

Two reviewers (EI and HI) independently assessed the risk of bias using version 2 of the Cochrane Risk-of-Bias Tool for Randomized Trials (RoB 2) (14). Their quality was evaluated using the Risk of Bias in Non-randomized Studies of Interventions (ROBINS-I) tool (15). Discrepancies were resolved by consensus or by a third reviewer (HO). Risk-of-bias plots were generated using the robvis web application (16).

### 2.7 Measurement of treatment effects

For dichotomous variables, including mortality, neurological outcomes, rebleeding, thromboembolism, DCI, and hydrocephalus, random effects models were used to calculate the relative risk (RR) with a 95% confidence interval (CI). Adverse events, defined by the original authors, were summarized descriptively but were excluded from the meta-analysis.

### 2.8 Data synthesis and statistical analyses

All analyses were conducted using the Review Manager software (RevMan 5.4.2; Nordic Cochrane Center, Cochrane Collaboration, Copenhagen, Denmark) to calculate pooled estimates and generate forest plots. Missing data not reported in the published manuscripts were requested by the original authors. For dichotomous data, the ITT analysis assumed that participants lost to follow-up before the event did not experience the event. Missing continuous data were not imputed, following the Cochrane Handbook (17). When only the median and interquartile range were reported, the median was converted to the mean and standard deviation using the Cochrane Handbook methods (17). Meta-analyses used data extracted from the original studies.

Statistical heterogeneity was assessed by forest plot inspection and I^2^ statistics. Heterogeneity was categorized as follows: 0–40% (likely unimportant), 30–60% (moderate heterogeneity), 50–90% (substantial heterogeneity), and 75–100% (considerable heterogeneity) (17). The Cochrane Chi^2^ test (Q-test) to assess the I^2^ statistic, with *p-*values < 0.10 considered statistically significant. Subgroup analyses of the primary outcomes in the older age group were conducted according to the protocol for substantial heterogeneity (I^2^ > 50%).

### 2.9 Reporting bias assessment

Clinical trial registries (ClinicalTrials.gov and WHO ICTRP) and literature were extensively searched for unpublished trials. Outcome reporting bias was assessed by comparing the trial protocols with published outcomes. Publication bias was evaluated using funnel plot inspection and Egger’s test, with *p* < 0.10 indicating statistical significance.

### 2.10 Subgroup analyses

Subgroup analyses examined the impact of TXA administration duration, categorized as ultra-early short-term use (≤24 h), short-term use (≤72 h), and long-term use (>72 h).

### 2.11 Sensitivity analyses

Sensitivity analyses assessed heterogeneity, evaluated the impact of bias on effect estimates, and excluded studies with a high risk of bias from the primary outcome analyses.

### 2.12 Certainty of evidence

The outcomes were summarized and their certainty of evidence was determined using the GRADEpro tool (McMaster University; Hamilton, ON, Canada), considering the risk of bias, imprecision, inconsistency, indirectness, and publication bias. Several NRSIs had a serious to critical risk of bias; therefore, the RCT and NRSI results were analyzed separately. Evidence from the included studies was listed, and outcome strength was evaluated according to the GRADE approach (18). GRADE recommendations were based solely on RCT data.

### 2.13 Difference between protocol and review

A subgroup analysis for DCI prevention was not conducted because only two studies explicitly reported the implementation of such measures (3, 5). Sensitivity analyses using imputed statistics for the primary outcome were not performed because no studies used imputed data. Analyses limited to participants with complete data were also not conducted, as none of the studies reported incomplete data.

## 3 Results

### 3.1 Search results

**Figure 1:**
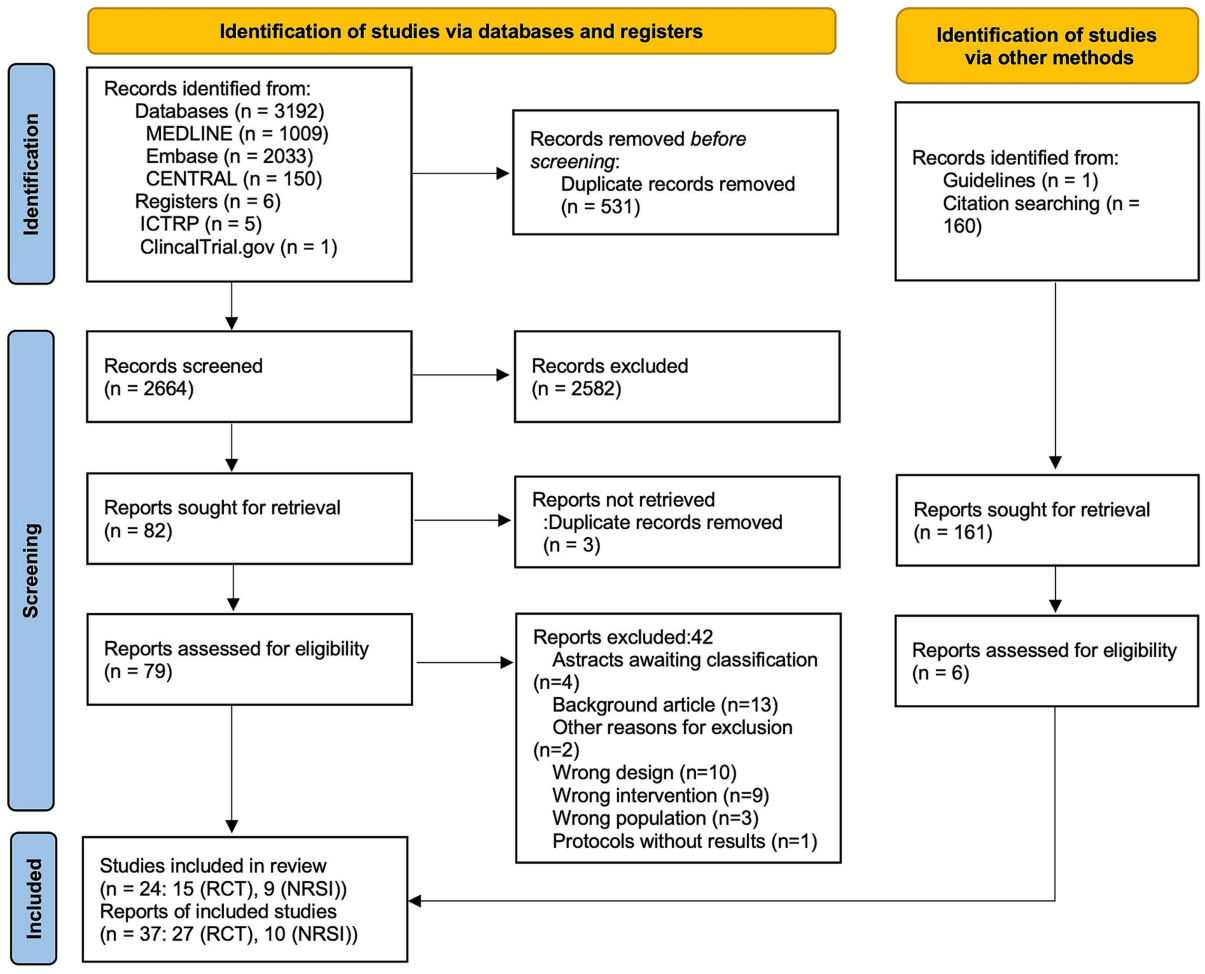
PRISMA 2020 flow diagram. CENTRAL: Cochrane Central Register of Controlled Trials; ICTRP, International Clinical Trials Registry Platform; RCTs, Randomized Controlled Trials.

A total of 3,198 records were screened through May 2024, and 79 studies underwent full-text review (Figure 1). Ultimately, 15 RCTs (3,109 patients) and nine NRSIs (1,506 patients) from 37 reports were included in this review (Figure 1; Table 1). The reasons for excluding 42 reports from the second screening are listed in Supplementary Table S3. Fodstad 1980 (19) included two studies and was analyzed separately: one with 46 unique participants and the other overlapping with the Fodstad 1981–2 (20) publication (59 participants). Among the RCTs, one study (5) administered treatments within 24 h, nine studies (19–27) included treatments administered within 72 h, and four studies (3, 4, 28, 29) included treatments administered beyond 72 h. Among NRSI, one study (30) administered treatment within 24 h of onset, three studies (31–33) within 72 h, and two studies beyond 72 h (34, 35), whereas the timing of administration was unclear in four studies (Supplementary Table S4) (35–38).

**Table 1:** Characteristics of the included RCTs.

References	Country	Study types	Number of patients; total (intervention/control)	Intervention (drug dosage, route, duration)	Comparator	Time from symptom onset to treatment (h)	Ischemia prophylaxis
van Rossum et al. (28)	The Netherlands	Double-blind	51 (26/25)	TXA 1 g/6 h, intravenously, 10 days or until surgery	Placebo (saline)	Within 0–14 days	Unclear
Chandra (4)	England	Double-blind	39 (20/19)	TXA 1 g/4 h, intravenously, 14–21 days	Placebo (saline)	Within 7 days	Unclear
Maurice-Williams (29)	England	Non-blinded	50 (25/25)	TXA 1.5 g/6 h, intravenously, 6 weeks or until operation, followed by 1.5 g/6 h, orally, 6 weeks or until operation	Standard therapy	Within 96 h	Unclear
Kaste and Ramsay (21)	Finland	Double-blind	64 (32/32)	TXA 1 g/4 h, intravenously, until surgery or at least 21 days if no surgery	Placebo (saline)	Within 72 h	Unclear
Fodstad (19)	Sweden	Double-blind	46 (23/23)	TXA 1 g/4 h, intravenously, 7 days, followed by 1 g/6 h, intravenously, day 8–35, then 1 g/8 h, orally, 6th week	Standard therapy	Within 72 h	Unclear
Fodstad et al. (20)	Sweden	Non-blinded	41 (21/20)	TXA 1 g/4 h, intravenously, 7 days, followed by 1 g/6 h, intravenously, day 8–28	Standard therapy	Unclear	Unclear
Fodstad et al. (20)	Sweden	Non-blinded	59 (30/29)	TXA 1 g/4 h, intravenously, 7 days, then 1 g/6 h, intravenously, day 8–21, followed by 1.5 g/6 h, orally, 3–6th weeks	Standard therapy	Within 72 h	Unclear
Vermeulen et al. (22)	The Netherlands	Double-blind	479 (241/238)	TXA 1 g/4 h, intravenously, 7 days, followed by 1 g/6 h, intravenously, day 8–28	Placebo (saline)	Within 72 h	Unclear
Hijdra et al. (23)	The Netherlands	Non-blinded	176 (88/88)	TXA 6 g/day, intravenously, 7 days, followed by 4 g/day, intravenously, day 8–28	Standard therapy	Within 72 h	Unclear
Tsementzis et al. (25)	England	Double-blind	100 (50/50)	TXA 1.5 g/4 h, intravenously, 7 days, followed by 1.5 g/4 h, orally, day 8–28	Standard therapy	Within 72 h	Unclear
Menzies et al. (24)	England	Double-blind	31 (17/14)	TXA 9 g/day, intravenously, 7 days, followed by 9 g/day, orally, day 8–21	Standard therapy	Within 72 h	Unclear
Tsementzis et al. (26)	England	Double-blind	19 (9/10)	TXA 1.5 g/4 h, intravenously, 7 days, followed by 1.5 g/4 h, orally, day 8–28	Standard therapy	Within 72 h	Unclear
Roos and STAR Study Group (3)	The Netherlands	Double-blind	462 (229/233)	TXA 1 g bolus + 1 g/4 h, intravenously, 7 days, followed by 1.5 g/6 h, other, day 8–21	Standard therapy	Within 96 h	Administer nimodipine 60 mg/4 h, orally, 3 weeks
Hillman et al. (27)	Sweden	Open-label	505 (254/251)	TXA 1 g bolus + 1 g/6 h, intravenously, 2 days	Standard therapy	within 48 h	Unclear
Post et al. (5)	The Netherlands	Open-label	955 (480/475)	TXA 1 g bolus + 1 g/8 h, intravenously, 1 day or until aneurysm treatment	Standard therapy	Within 24 h	Nimodipine and normovolemia

NI; no information, TXA; tranexamic acid.

### 3.2 Risk of bias of included studies

**Figure 2:**
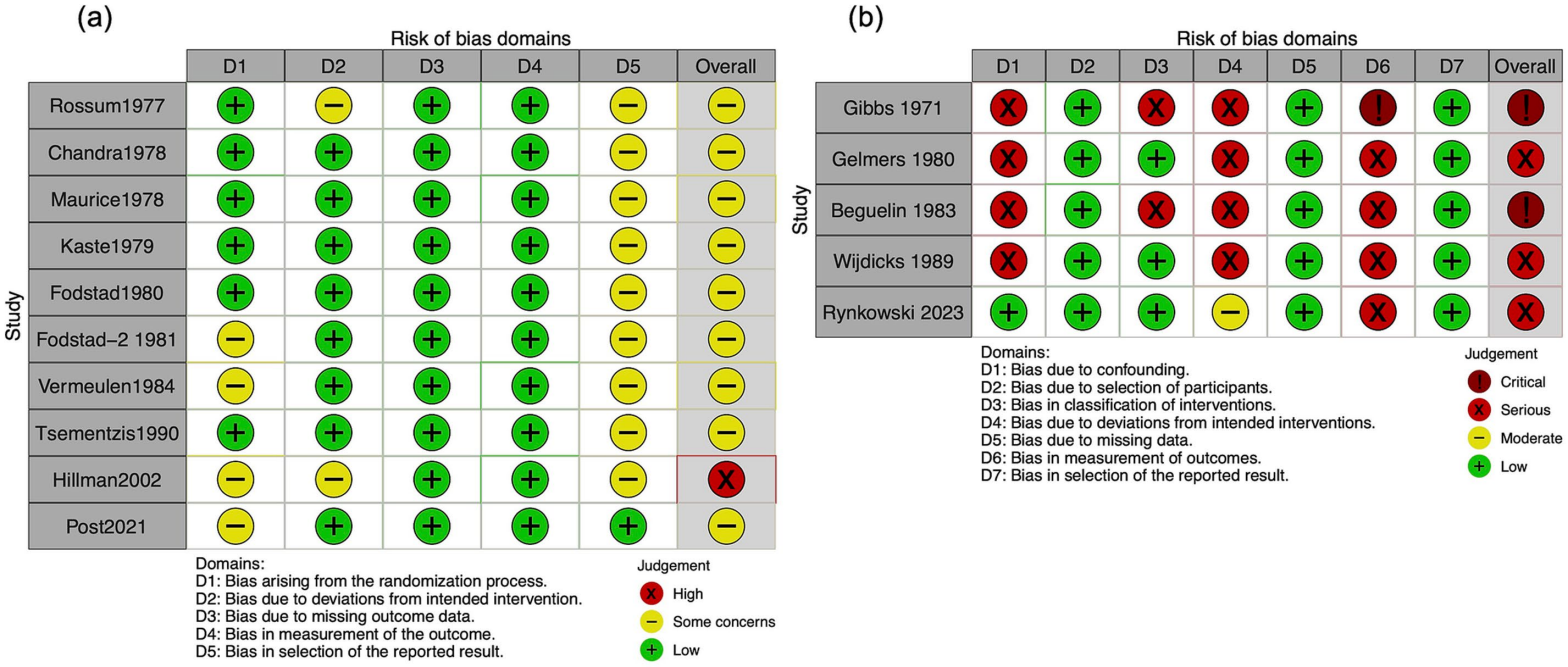
Risk of bias in the included studies evaluating the mortality. **(a)** Randomized controlled trials. **(b)** Non-randomized studies of interventions.

The Cochrane risk-of-bias tool indicated high concern for the included RCTs. The ROBINS-I tool identified a moderate to critical risk of bias in the included NRSI. The detailed assessment results are shown in Figure 2 and Supplementary Figures S1–S5.

### 3.3 Primary outcomes

#### 3.3.1 Mortality

**Figure 3:**
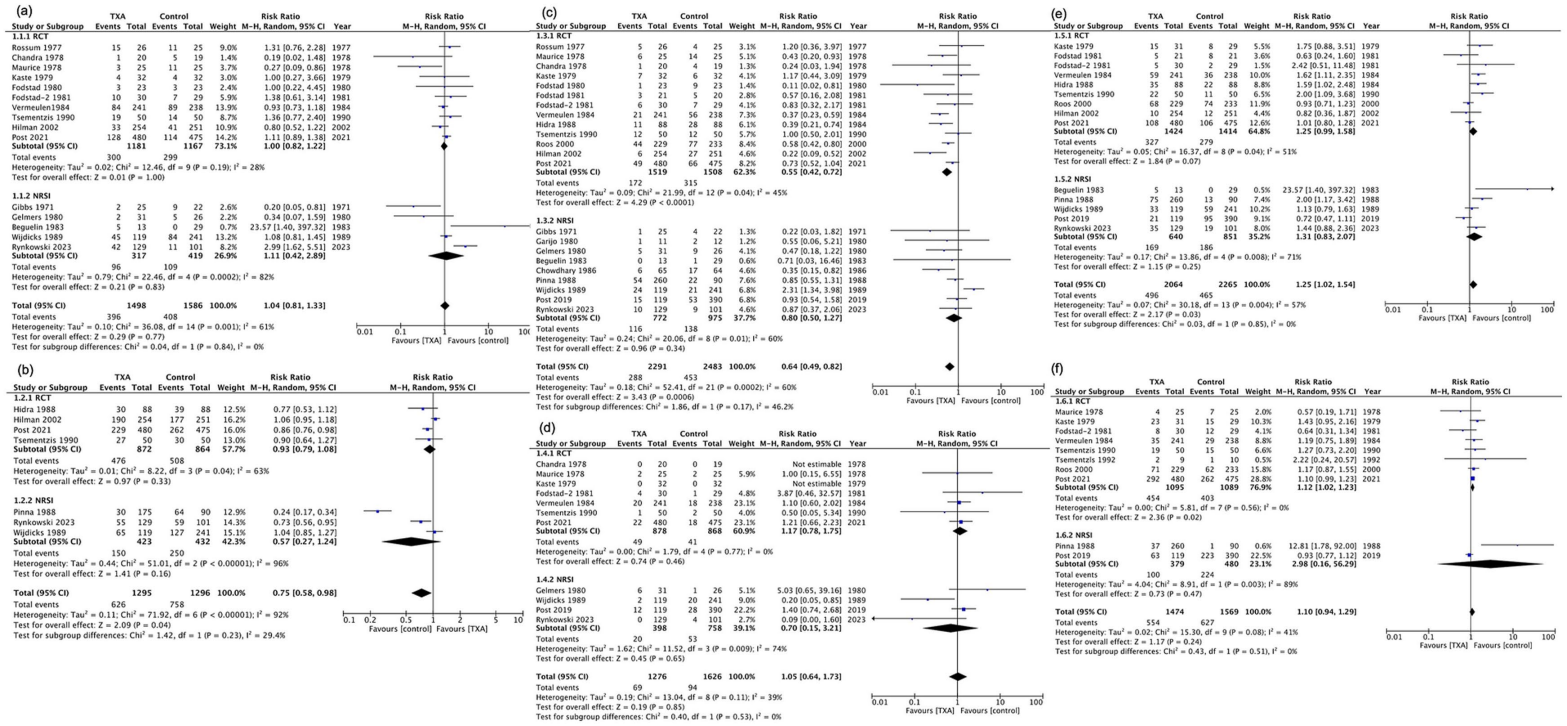
Forest plot of primary and secondary outcomes. **(a)** Mortality. **(b)** Good neurological outcomes. **(c)** Rebleeding. **(d)** Thromboembolism. **(e)** Delayed cerebral ischemia. **(f)** Hydrocephalus. CI, confidence interval; df, degrees of freedom; M–H, Mantel–Haenszel; SD, standard deviation; TXA, tranexamic acid.

A total of 10 RCTs (2,348 participants) were evaluated in our meta-analysis, showing that TXA likely has little to no difference in mortality (RR, 1.00; 95% CI, 0.82–1.22; I^2^ = 28%; moderate certainty; Table 2). Although included NRSIs exhibited high heterogeneity, they yielded findings consistent with RCTs (RR, 1.11; 95% CI, 0.42–2.89; I^2^ = 82%; serious to critical risk of bias; Figures 2, 3).

**Table 2:** Summary of findings.

Outcomes	Anticipated absolute effects^*^ (95% CI)		Relative effect (95% CI)	No of participants (studies)	Certainty of the evidence (GRADE)	Comments
	Risk with control	Risk with TXA				
Mortality	256 per 1,000	256 per 1,000 (210 to 313)	RR 1.00 (0.82 to 1.22)	2,348 (10 RCTs)	⨁⨁◯◯ Moderate^a^	TXA likely has little to no difference in mortality
Neurological outcomes	588 per 1,000	547 per 1,000 (464 to 635)	RR 0.93 (0.79 to 1.08)	1,736 (4 RCTs)	⨁⨁⨁◯ Moderate^a^	TXA likely results have little to no difference in good neurological outcome
Rebleeding	209 per 1,000	115 per 1,000 (88 to 150)	RR 0.55 (0.42 to 0.72)	3,027 (13 RCTs)	⨁⨁⨁◯ Moderate^a^	TXA likely reduces rebleeding
Thromboembolism	47 per 1,000	55 per 1,000 (37 to 83)	RR 1.17 (0.78 to 1.75)	1,746 (7 RCTs)	⨁⨁◯◯ Low^a^^,^^b^	TXA may have little to no difference in thromboembolism
DCI	197 per 1,000	247 per 1,000 (195 to 312)	RR 1.25 (0.99 to 1.58)	2,838 (9 RCTs)	⨁⨁◯◯ Low^a^^,^^b^	TXA may have little to no difference in DCI
Hydrocephalus	370 per 1,000	414 per 1,000 (377 to 455)	RR 1.12 (1.02 to 1.23)	2,184 (8 RCTs)	⨁⨁⨁◯ Moderate^a^	TXA probably increases hydrocephalus slightly

Summary of findings: Effect of TXA on outcomes in aneurysmal SAH from RCTs: Patient: Patients with aneurysmal SAH. Setting: Emergency departments, intensive care units, and hospital wards. Intervention: TXA. Comparison: Control (placebo or standard therapy). *The risk in the intervention group (and its 95% confidence interval) is based on the assumed risk in the comparison group and the relative effect of the intervention (and its 95% CI). CI, confidence interval; DCI, delayed cerebral ischemia; RCT, randomized controlled trial; RR: risk ratio; SAH, subarachnoid hemorrhage; TAX, tranexamic acid. GRADE working group grades of evidence: High certainty: We are very confident that the true effect lies close to that of the estimate of the effect. Moderate certainty: We are moderately confident in the effect estimate: the true effect is likely to be close to the estimate of the effect; however, there is a possibility that it is substantially different. Low certainty: Our confidence in the effect estimate is limited: the true effect may be substantially different from the estimate of the effect. Very low certainty: We have very little confidence in the effect estimate: the true effect is likely to be substantially different from the estimate of effect.

a Downgraded one point due to the risk of bias, which consists only of some concerns and high concerns.

b Downgraded one point because the 95% confidence interval (CI) straddles the clinical threshold.

### 3.4 Secondary outcomes

#### 3.4.1 Neurological outcome

Four RCTs (1,736 participants) demonstrated that TXA likely has little to no difference in good neurological outcome (RR, 0.93; 95% CI, 0.79–1.08; I (2) = 63%; moderate certainty; Table 2; Figure 3; Supplementary Figure S1). Three NRSI (855 participants) yielded consistent results but exhibited high heterogeneity (RR, 0.75; 95% CI, 0.58–0.98; I^2^ = 96%; serious risk of bias; Figure 3; Supplementary Figure S1). Five of the seven studies used GOS-E (23, 25, 27, 32, 34), while two studies assessed outcomes using mRS (5, 33).

#### 3.4.2 Rebleeding

Thirteen RCTs (3,027 participants) demonstrated that TXA likely reduces rebleeding (RR, 0.55; 95% CI, 0.42–0.72; I^2^ = 45%; moderate certainty; Table 2; Figure 3; Supplementary Figure S2). Nine NRSIs (1,747 participants) were pooled (RR, 0.80; 95% CI, 0.50–1.27; I^2^ = 60%; moderate to critical risk of bias). Both the RCTs and NRSIs displayed a similar direction of effect (Figure 3; Supplementary Figure S2).

#### 3.4.3 Thromboembolism

Seven RCTs (1,746 participants) demonstrated that TXA may have little to no difference in thromboembolism (RR, 1.17; 95% CI, 0.78–1.75; I^2^ = 0%; low certainty; Table 2, Figure 3; Supplementary Figure S3). Four NRSIs (1,156 participants) were pooled (RR, 0.80; 95% CI, 0.70–3.21; I^2^ = 74%; serious to critical risk of bias). Although the RCTs and NRSIs displayed opposite effects, the NRSI results had a serious or critical bias and low reliability (Figure 3; Supplementary Figure S3).

#### 3.4.4 DCI

Nine RCTs (2,838 participants) demonstrated that TXA may have little to no difference in DCI (RR, 1.25; 95% CI, 0.99–1.58; I^2^ = 51%; low certainty; Table 2; Figure 3; Supplementary Figure S4). Five NRSIs (1,491 participants) were pooled (RR, 1.31; 95% CI, 0.83–2.07; I^2^ = 71%; serious to critical risk of bias). Both the RCTs and NRSIs displayed a similar direction of effect (Figure 3; Supplementary Figure S4).

#### 3.4.5 Hydrocephalus

Eight RCTs (2,184 participants) demonstrated that TXA probably increases hydrocephalus slightly (RR, 1.12; 95% CI, 1.02–1.23; I^2^ = 0%; moderate certainty; Table 2, Figure 3; Supplementary Figure S5). Two NRSIs (859 participants) were pooled (RR: 2.98; 95% CI, 0.16–56.29; I^2^ = 89%; moderate to serious risk of bias). Unlike the NRSIs, the pooled RR in the RCTs favored the control group (Figure 3; Supplementary Figure S5). Three studies reported a rebleeding RR < 1 and a hydrocephalus RR < 1; four studies reported a rebleeding RR < 1 and a hydrocephalus RR ≥ 1; and two studies reported both rebleeding and hydrocephalus RRs ≥ 1 (Supplementary Figure S6).

#### 3.4.6 Adverse events

Only two studies reported adverse events other than primary and secondary outcomes. Two studies reported delirium, with incidence ranging from 2.0 to 13.5% in the TXA group and from 10.0 to 12.8% in the control group (5, 25). One study reported the following complication rates for TXA versus controls: severe hyponatremia (2.4% vs. 2.0%), pneumonia (12.6% vs. 14.6%), infectious meningitis (7.4% vs. 6.7%), urinary tract infection (9.6% vs. 9.1%), seizures (12.0% vs. 8.9%), and Terson syndrome (3.9% vs. 3.6%) (5).

### 3.5 Reporting bias assessment

Funnel plots (Supplementary Figures S7–S12) did not indicate publication bias. Egger’s test was performed for mortality and rebleeding, as both outcomes included more than 10 RCTs; however, no evidence of publication bias was observed (*p* = 0.32 and 0.58, respectively).

### 3.6 Subgroup analysis

In the RCTs, a subgroup analysis of the primary outcome was conducted based on the duration of TXA administration. One study (5) administered TXA within 24 h, six studies (19–22, 25, 27) within 72 h, and three studies (4, 28, 29) after 72 h. The pooled results remained consistent across all subgroups, showing no differences between the TXA and control groups (Table 1; Supplementary Figure S13).

### 3.7 Sensitivity analysis

One study with a high risk of bias was removed from the primary analysis. This exclusion did not affect the results, which showed no difference between the TXA and control groups (Supplementary Figure S14).

## 4 Discussion

This is the first meta-analysis to rigorously compare RCTs and NRSIs on TXA use for SAH. Fifteen RCTs (3,109 participants) were included to provide GRADE-based evidence on TXA’s efficacy and safety of TXA. Additionally, nine NRSIs (1,506 participants) were analyzed. Overall, TXA administration, regardless of the timing or method, did not affect mortality, favorable neurological outcomes, thromboembolism, or DCI. However, it likely reduced the risk of rebleeding while slightly increasing the incidence of hydrocephalus. As rebleeding occurs in less than 4% of patients (39), routine TXA use offers no apparent benefit. However, its use should be considered in patients with a high risk of rebleeding.

TXA may be beneficial when early surgical intervention is not feasible; however, its risks, including hydrocephalus, should be carefully considered. Therefore, routine administration is not supported. Although older studies used TXA for 3–4 weeks post-SAH (3, 26), recent trends favor its administration within 1–2 days or until surgery (5, 27). Subgroup analyses demonstrated no benefit from ultra-early (≤24 h) or short-term (≤72 h) administration, whereas prolonged use (>72 h) showed only a modest trend toward reduced rebleeding, with substantial heterogeneity. The widespread adoption of early, definitive interventions such as surgical clipping or endovascular coiling may have contributed to more consistent rebleeding prevention. Despite methodological variations, including the use of two different neurological outcome scales (mRS and GOS-E) across studies, recent studies have demonstrated diminishing differences in rebleeding and mortality over time between the TXA and control groups. Rebleeding-related mortality is approximately 80% (39) and because TXA reduced rebleeding incidence (RR ≈ 0.55), its use may be considered in high-risk patients, such as those with (1) increasing aneurysm size, (2) worsening neurological deficits, (3) angiography within 3 h of bleeding, (4) sentinel hemorrhage, and (5) loss of consciousness at initial bleed (39). Future studies should evaluate the impact of TXA in these high-risk populations to determine its role in reducing rebleeding, mortality, and neurological complications.

Unlike previous SR/MA, this study applied GRADE assessments to different TXA-related outcomes with certainty ranging from low to moderate. Recent SR/MA have faced limitations, including the inclusion of non-SAH hemorrhages (40), misclassification of observational studies as RCTs (9, 10), lack of GRADE assessments (41), inconsistencies between GRADE assessments and recommendations (42), and duplicate inclusion of the same study by Foodstad as two separate studies (43). These methodological issues have resulted in an unclear understanding of the role of TXA in SAH. By addressing these limitations, this study provides a more objective assessment. Specifically, we excluded non-SAH hemorrhages based solely on GRADE assessments of RCTs to minimize confounding and incorporated NRSIs to enhance the comprehensiveness of our analysis. The application of these methodological refinements established that the certainty of the GRADE assessments for different outcomes ranged from low to moderate. Consequently, this review included the largest number of RCTs and observational studies to date and provided the most detailed effect size estimates for TXA in SAH management.

This study had several limitations. First, several of the included studies were outdated, with longer intervals between SAH onset and study enrollment and prolonged TXA administration. However, our sensitivity analysis of recent RCTs demonstrated that studies with shorter enrollment and administration periods demonstrated similar trends in mortality, neurological outcomes, and rebleeding. Additionally, modern management strategies, such as venous thromboembolism prophylaxis and spinal drains for hydrocephalus prevention (30), may have influenced the comparability between older and recent studies. Second, seizure reporting was limited. Risk factors for TXA-associated seizures include renal impairment, female sex, epilepsy history, age > 70 years, and high-dose administration (>50 mg/kg) (44). Among the included studies, only one RCT (5) reported seizure, suggesting insufficient power to detect complications or difficulty in differentiating TXA-induced seizures from SAH-related seizures. Future studies should specifically investigate the seizure risk to elucidate the safety profile of TXA in SAH management. Third, a major limitation was the insufficient reporting of detailed patient admission status and TXA administration protocols across the included studies. Only four studies (16.7%) reported Fisher grades, nine (37.5%) reported Hunt-Hess grades, and two (8.3%) reported World Federation of Neurosurgical Societies (WFNS) grades, indicating substantial gaps in baseline clinical characterization. Furthermore, substantial heterogeneity in TXA administration parameters—including bolus use, infusion rate, total dosage, and timing relative to aneurysm treatment—was observed across studies, limiting comparability and synthesis of findings. This lack of granular data precluded a more refined analysis to establish objective criteria for patient selection and to elucidate the therapeutic balance between rebleeding prevention and hydrocephalus risk. Future research should prioritize standardized and detailed reporting of both admission characteristics and TXA protocols. In particular, dose–response meta-analyses incorporating these variables may help define optimal dosing strategies tailored to patient-specific risk profiles. Fourth, although CT was the primary modality for diagnosing rebleeding and hydrocephalus, other approaches (angiography, CSF analysis, autopsy, and clinical deterioration) were also used, which may have contributed to heterogeneity. We assessed outcome-measurement bias using RoB 2 and ROBINS-I, but future studies should standardize diagnostic criteria for TXA evaluation in SAH.

## 5 Conclusion

This meta-analysis reveals that TXA likely has little to no impact on mortality; however, it probably reduces rebleeding while slightly increasing hydrocephalus risk, with moderate-certainty evidence. In clinical practice, TXA should be reserved for selected patients with a high risk of rebleeding.

## Data Availability

The datasets presented in this study can be found in online repositories. The names of the repository/repositories and accession number(s) can be found in the article/Supplementary material.
