# Correction: Efficacy and safety of tranexamic acid administration for subarachnoid hemorrhage: a systematic review and meta-analysis

**DOI:** 10.3389/fneur.2025.1754088

**Published:** 2025-12-15

**Authors:** Eriya Imai, Hiroshi Ito, Hiromu Okano, Akihiko Inoue, Takero Terayama, Hiroshi Okamoto, Toru Hifumi, Yoshihisa Fujimoto, Gaku Fujiwara, Yasuhiro Kuroda

**Affiliations:** 1 Division of Anesthesia, Mitsui Memorial Hospital, Chiyoda-ku, Tokyo, Japan; 2 Department of Traumatology and Acute Critical Medicine, Osaka University Graduate School of Medicine, Osaka, Japan; 3 Department of Critical Care Medicine, St. Luke's International Hospital, Chuo-ku, Tokyo, Japan; 4 Department of Emergency and Critical Care Medicine, Hyogo Emergency Medical Center, Chuo-ku, Kobe, Japan; 5 Department of Emergency, Self Defense Forces Central Hospital, Setagaya-Ku, Tokyo, Japan; 6 Department of Emergency and Critical Care Medicine, St. Luke's International Hospital, Chuo-ku, Tokyo, Japan; 7 Department of Emergency and Critical Care Medicine, St. Marianna University School of Medicine Hospital, Miyamae-ku, Kawasaki, Japan; 8 Department of Management of Technology and Intellectual Property, School of Public Health, Kyoto University, Kyoto, Japan; 9 Department of Emergency, Disaster, and Critical Care Medicine, Faculty of Medicine, Kagawa University, Miki, Japan

**Keywords:** meta-analysis, subarachnoid hemorrhage, systematic review, tranexamic acid, rebleeding

In the published article, author Hiroshi Ito was erroneously omitted as an equal contributing and co-first author.

The original version of this article has been updated.

