# Supplementary material for: Efficacy and safety of tranexamic acid administration for subarachnoid hemorrhage: a systematic review and meta-analysis
Source: Front Neurol. 2025 Jun 17;16:1617817. doi: 10.3389/fneur.2025.1617817 (PMC12209260; doi:10.3389/fneur.2025.1617817)
Supplement: Supplementary file 1 [file Table_1.docx]

**Supplementary materials**

Table of Contents

[Table S1. PRISMA checklist 2](#_Toc198908547)

[Table S2. Search strategy 5](#_Toc198908548)

[Table S3. Excluded studies 8](#_Toc198908549)

[Table S4: Characteristics of included NRSI 12](#_Toc198908550)

[Figure S1. Risk of bias in the included studies evaluating the neurological outcome. 14](#_Toc198908551)

[Figure S2. Risk of bias in the included studies evaluating the rebleeding. 15](#_Toc198908552)

[Figure S3. Risk of bias in the included studies evaluating thromboembolism. 16](#_Toc198908553)

[Figure S4. Risk of bias in the included studies evaluating the DCI. 17](#_Toc198908554)

[Figure S5. Risk of bias in the included studies evaluating the hydrocephalus. 18](#_Toc198908555)

[Figure S6. Risk-benefit diagram plotting for rebleeding and hydrocephalus. 19](#_Toc198908556)

[Figure S7. Funnel plot assessing publication bias for the mortality. 19](#_Toc198908557)

[Figure S8. Funnel plot assessing publication bias for the neurological outcome. 20](#_Toc198908558)

[Figure S9. Funnel plot assessing publication bias for the rebleeding. 20](#_Toc198908559)

[Figure S10. Funnel plot assessing publication bias for the thromboembolism. 20](#_Toc198908560)

[Figure S11. Funnel plot assessing publication bias for DCI. 21](#_Toc198908561)

[Figure S12. Funnel plot assessing publication bias for hydrocephalus. 21](#_Toc198908562)

[Figure S13. Forest plot of subgroup analysis in mortality. 22](#_Toc198908563)

[Figure S14. Forest plot of sensitivity analysis in mortality. 22](#_Toc198908564)

# **Table S1.** PRISMA checklist

| **Section and Topic** | **Item #** | **Checklist item** | **Location where item is reported** |
| --- | --- | --- | --- |
| **TITLE** | | |  |
| Title | 1 | Identify the report as a systematic review. | TITLE |
| **ABSTRACT** | | |  |
| Abstract | 2 | See the PRISMA 2020 for Abstracts checklist. | ABSTRACT |
| **INTRODUCTION** | | |  |
| Rationale | 3 | Describe the rationale for the review in the context of existing knowledge. | INTRODUCTION |
| Objectives | 4 | Provide an explicit statement of the objective(s) or question(s) the review addresses. | INTRODUCTION |
| **METHODS** | | |  |
| Eligibility criteria | 5 | Specify the inclusion and exclusion criteria for the review and how studies were grouped for the syntheses. | METHODS |
| Information sources | 6 | Specify all databases, registers, websites, organisations, reference lists and other sources searched or consulted to identify studies. Specify the date when each source was last searched or consulted. | METHODS |
| Search strategy | 7 | Present the full search strategies for all databases, registers and websites, including any filters and limits used. | Online Resource |
| Selection process | 8 | Specify the methods used to decide whether a study met the inclusion criteria of the review, including how many reviewers screened each record and each report retrieved, whether they worked independently, and if applicable, details of automation tools used in the process. | METHODS |
| Data collection process | 9 | Specify the methods used to collect data from reports, including how many reviewers collected data from each report, whether they worked independently, any processes for obtaining or confirming data from study investigators, and if applicable, details of automation tools used in the process. | METHODS |
| Data items | 10a | List and define all outcomes for which data were sought. Specify whether all results that were compatible with each outcome domain in each study were sought (e.g. for all measures, time points, analyses), and if not, the methods used to decide which results to collect. | METHODS |
|  | 10b | List and define all other variables for which data were sought (e.g. participant and intervention characteristics, funding sources). Describe any assumptions made about any missing or unclear information. | METHODS |
| Study risk of bias assessment | 11 | Specify the methods used to assess risk of bias in the included studies, including details of the tool(s) used, how many reviewers assessed each study and whether they worked independently, and if applicable, details of automation tools used in the process. | METHODS |
| Effect measures | 12 | Specify for each outcome the effect measure(s) (e.g. risk ratio, mean difference) used in the synthesis or presentation of results. | METHODS |
| Synthesis methods | 13a | Describe the processes used to decide which studies were eligible for each synthesis (e.g. tabulating the study intervention characteristics and comparing against the planned groups for each synthesis (item #5)). | METHODS |
|  | 13b | Describe any methods required to prepare the data for presentation or synthesis, such as handling of missing summary statistics, or data conversions. | METHODS |
|  | 13c | Describe any methods used to tabulate or visually display results of individual studies and syntheses. | METHODS |
|  | 13d | Describe any methods used to synthesize results and provide a rationale for the choice(s). If meta-analysis was performed, describe the model(s), method(s) to identify the presence and extent of statistical heterogeneity, and software package(s) used. | METHODS |
|  | 13e | Describe any methods used to explore possible causes of heterogeneity among study results (e.g. subgroup analysis, meta-regression). | METHODS |
|  | 13f | Describe any sensitivity analyses conducted to assess robustness of the synthesized results. | METHODS |
| Reporting bias assessment | 14 | Describe any methods used to assess risk of bias due to missing results in a synthesis (arising from reporting biases). | METHODS |
| Certainty assessment | 15 | Describe any methods used to assess certainty (or confidence) in the body of evidence for an outcome. | METHODS |
| **RESULTS** | | |  |
| Study selection | 16a | Describe the results of the search and selection process, from the number of records identified in the search to the number of studies included in the review, ideally using a flow diagram. | RESULTS |
|  | 16b | Cite studies that might appear to meet the inclusion criteria, but which were excluded, and explain why they were excluded. | Online Resource |
| Study characteristics | 17 | Cite each included study and present its characteristics. | RESULTS Table 1 |
| Risk of bias in studies | 18 | Present assessments of risk of bias for each included study. | Figure2, Online Resource |
| Results of individual studies | 19 | For all outcomes, present, for each study: (a) summary statistics for each group (where appropriate) and (b) an effect estimate and its precision (e.g. confidence/credible interval), ideally using structured tables or plots. | RESULTS |
| Results of syntheses | 20a | For each synthesis, briefly summarise the characteristics and risk of bias among contributing studies. | RESULTS |
|  | 20b | Present results of all statistical syntheses conducted. If meta-analysis was done, present for each the summary estimate and its precision (e.g. confidence/credible interval) and measures of statistical heterogeneity. If comparing groups, describe the direction of the effect. | RESULTS |
|  | 20c | Present results of all investigations of possible causes of heterogeneity among study results. | RESULTS |
|  | 20d | Present results of all sensitivity analyses conducted to assess the robustness of the synthesized results. | RESULTS, Online Resource |
| Reporting biases | 21 | Present assessments of risk of bias due to missing results (arising from reporting biases) for each synthesis assessed. | RESULTS |
| Certainty of evidence | 22 | Present assessments of certainty (or confidence) in the body of evidence for each outcome assessed. | Table 2 |
| **DISCUSSION** | | |  |
| Discussion | 23a | Provide a general interpretation of the results in the context of other evidence. | DISCUSSION |
|  | 23b | Discuss any limitations of the evidence included in the review. | DISCUSSION |
|  | 23c | Discuss any limitations of the review processes used. | DISCUSSION |
|  | 23d | Discuss implications of the results for practice, policy, and future research. | DISCUSSION |
| **OTHER INFORMATION** | | |  |
| Registration and protocol | 24a | Provide registration information for the review, including register name and registration number, or state that the review was not registered. | ABSTRACT, METHODS |
|  | 24b | Indicate where the review protocol can be accessed, or state that a protocol was not prepared. | METHODS |
|  | 24c | Describe and explain any amendments to information provided at registration or in the protocol. | METHODS |
| Support | 25 | Describe sources of financial or non-financial support for the review, and the role of the funders or sponsors in the review. | DISCLOSURE |
| Competing interests | 26 | Declare any competing interests of review authors. | DISCLOSURE |
| Availability of data, code and other materials | 27 | Report which of the following are publicly available and where they can be found: template data collection forms; data extracted from included studies; data used for all analyses; analytic code; any other materials used in the review. | DISCLOSURE |

*From:*  Page MJ, McKenzie JE, Bossuyt PM, Boutron I, Hoffmann TC, Mulrow CD, et al. The PRISMA 2020 statement: an updated guideline for reporting systematic reviews. BMJ 2021;372:n71. doi: 10.1136/bmj.n71

# **Table S2.** Search strategy

**MEDLINE via Pubmed** (accessed on May 30, 2024)

("Antifibrinolytic Agents"[MeSH Terms] OR "Antifibrinolytic Agents"[Pharmacological Action] OR "Tranexamic Acid"[MeSH Terms] OR "Aminocaproic Acid"[Text Word] OR ("TXA"[Text Word] OR "tranexamic"[Text Word] OR "4 aminomethylcyclohexanecarboxylic acid"[Text Word] OR "amca"[Text Word] OR "AMCHA"[Text Word] OR "amikapron"[Text Word] OR "aminomethyl cyclohexane carboxylic acid"[Text Word] OR "aminomethyl cyclohexanecarboxylic acid"[Text Word] OR "aminomethylcyclohexanecarboxylic acid"[Text Word] OR "amstat"[Text Word] OR "anvitoff"[Text Word] OR "cl 65336"[Text Word] OR "cyclocapron"[Text Word] OR "cyclokapron"[Text Word] OR "cyklocapron"[Text Word] OR "cyklokapron"[Text Word] OR "exacyl"[Text Word] OR "frenolyse"[Text Word] OR "hexakapron"[Text Word] OR "lysteda"[Text Word]) OR ("tranex"[Text Word] OR "tranexam"[Text Word] OR "tranexanic acid"[Text Word] OR "trans 4 aminomethyl cyclohexanecarboxylic acid"[Text Word] OR "trans 4 aminomethylcyclohexane 1 carboxylic acid"[Text Word] OR "trans 4 aminomethylcyclohexane carboxylic acid"[Text Word] OR "trans 4 aminomethylcyclohexanecarboxylic acid"[Text Word] OR "trans amcha"[Text Word] OR "trans aminomethyl cyclohexane carboxylic acid"[Text Word] OR "trans aminomethylcyclohexane carboxylic acid"[Text Word] OR "trans aminomethylcyclohexanecarboxylic acid"[Text Word] OR "transexamic"[Text Word] OR "ugurol"[Text Word] OR "transamin*"[Text Word])) AND ("Subarachnoid Hemorrhage"[MeSH Terms] OR ("intracranial hemorrhages"[MeSH Terms:noexp] OR "Cerebral Hemorrhage"[MeSH Terms:noexp] OR "Hemorrhagic Stroke"[MeSH Terms]) OR ("Subarachnoid Hemorrhage"[Title/Abstract:~6] OR "Subarachnoid Hemorrhages"[Title/Abstract:~6] OR "Subarachnoid Haemorrhage"[Title/Abstract:~6] OR "Subarachnoid Haemorrhages"[Title/Abstract:~6] OR "Subarachnoid bleeding"[Title/Abstract:~6] OR "Subarachnoid Bleedings"[Title/Abstract:~6]) OR "cerebral aneurysm*"[Title/Abstract] OR "intracranial aneurysm*"[Title/Abstract] OR ("intracranial aneurysm"[MeSH Terms] AND "rupture, spontaneous"[MeSH Terms]) OR ("aneurysm, ruptured"[MeSH Terms:noexp] AND ("brain"[MeSH Terms] OR "Meninges"[MeSH Terms])))

**CENTRAL** (accessed on May 15, 2024)

#1 MeSH descriptor: [Subarachnoid Hemorrhage] explode all trees

#2 MeSH descriptor: [Intracranial Hemorrhages] this term only

#3 MeSH descriptor: [Cerebral Hemorrhage] this term only

#4 MeSH descriptor: [Hemorrhagic Stroke] explode all trees

#5 (subarachnoid NEAR h?emorrhage*):ti,ab,kw

#6 (subarachnoid NEAR bleeding*):ti,ab,kw

#7 #1 OR #2 OR #3 OR #4 OR #5 OR #6

#8 (cerebral aneurysm*):ti,ab,kw

#9 (intracranial aneurysm*):ti,ab,kw

#10 MeSH descriptor: [Intracranial Aneurysm] explode all trees

#11 MeSH descriptor: [Rupture, Spontaneous] explode all trees

#12 #10 AND #11

#13 MeSH descriptor: [Aneurysm, Ruptured] this term only

#14 MeSH descriptor: [Brain] explode all trees

#15 MeSH descriptor: [Meninges] explode all trees

#16 #14 or #15

#17 #13 and #16

#18 #7 or #8 or #9 or #12 or #17

#19 MeSH descriptor: [Antifibrinolytic Agents] explode all trees

#20 MeSH descriptor: [Tranexamic Acid] explode all trees

#21 (Aminocaproic Acid):ti,ab,kw

#22 (TXA OR tranexamic OR 4 aminomethylcyclohexanecarboxylic acid OR amca OR AMCHA OR amikapron OR aminomethyl cyclohexane carboxylic acid OR aminomethyl cyclohexanecarboxylic acid OR aminomethylcyclohexanecarboxylic acid OR amstat OR anvitoff OR cl 65336 OR cyclocapron OR cyclokapron OR cyklocapron OR cyklokapron OR exacyl OR frenolyse OR hexakapron OR lysteda):ti,ab,kw

#23 (tranex OR tranexam OR tranexanic acid OR trans 4 aminomethyl cyclohexanecarboxylic acid OR trans 4 aminomethylcyclohexane 1 carboxylic acid OR trans 4 aminomethylcyclohexane carboxylic acid OR trans 4 aminomethylcyclohexanecarboxylic acid OR trans amcha OR trans aminomethyl cyclohexane carboxylic acid OR trans aminomethylcyclohexane carboxylic acid OR trans aminomethylcyclohexanecarboxylic acid OR transexamic OR ugurol OR transamin*):ti,ab,kw

#24 #19 or #20 or #21 or #22 or #23

#25 #18 and #24

#26 #25 in Trials

**ICTRP** (accessed on May 15, 2024)

| #1 | (SAH OR Subarachnoid Hemorrhage) |
| --- | --- |
| #2 | (“Tranexamic Acid” OR TXA) |
| #3 | #1 AND #2 |

**ClinicalTrials.gov** (accessed on May 15, 2024)

| Condition or disease | (SAH OR Subarachnoid OR arachnoid) AND (Hemorrhage OR Bleeding) |
| --- | --- |
| Intervention | “Tranexamic Acid” OR TXA |

**EMBASE via ProQuest** (accessed on May 15, 2024)

(('subarachnoid hemorrhage'/de OR 'brain hemorrhage'/de OR (subarachnoid NEAR/6 h?emorrhage*):ti,ab,kw OR (subarachnoid NEAR/6 bleeding*):ti,ab,kw) OR ('intracranial aneurysm'/exp AND 'rupture'/de) OR ('aneurysm rupture'/de AND ('brain'/exp OR 'meninx'/exp))) AND ('antifibrinolytic agent'/exp OR (anti-fibrinolytic*:ti,ab,kw OR antifibrinolytic*:ti,ab,kw OR antifibrinolysin*:ti,ab,kw OR anti-fibrinolysin*:ti,ab,kw OR antiplasmin*:ti,ab,kw OR anti-plasmin*:ti,ab,kw OR ((plasmin NEAR/3 inhibitor*):ti,ab,kw)) OR (fibrinolysis NEAR/3 (prevent* OR inhib* OR antag*)):ti,ab,kw OR 'tranexamic acid'/de OR ('4 aminomethylcyclohexanecarboxylic acid':ti,ab,kw OR amca:ti,ab,kw OR amcha:ti,ab,kw) OR (amchafibrin:ti,ab,kw OR 'aminomethyl cyclohexanecarboxylic acid':ti,ab,kw) OR ('aminomethylcyclohexanecarboxylic acid':ti,ab,kw OR caprilon:ti,ab,kw) OR (cyclocapron:ti,ab,kw OR cyclokapron:ti,ab,kw OR cyklocapron:ti,ab,kw OR cyklokapron:ti,ab,kw OR exacyl:ti,ab,kw OR fibrinon:ti,ab,kw OR hexacapron:ti,ab,kw OR hexakapron:ti,ab,kw OR 'kabi 2161':ti,ab,kw) OR (spotof:ti,ab,kw OR tranex:ti,ab,kw OR tranexam:ti,ab,kw OR 'tranexamic acid':ti,ab,kw OR 'tranexanic acid':ti,ab,kw OR tranexic:ti,ab,kw) OR 'trans 4 aminomethylcyclohexanecarboxylic acid':ti,ab,kw OR ('trans aminomethylcyclohexanecarboxylic acid':ti,ab,kw OR transamin:ti,ab,kw OR 'transexamic acid':ti,ab,kw OR traxamic:ti,ab,kw OR ugurol:ti,ab,kw) OR 'aminocaproic acid derivative'/de OR (afibrin:ti,ab,kw OR amicar:ti,ab,kw OR 'amino caproic acid':ti,ab,kw OR 'aminocaproic acid':ti,ab,kw OR eaca:ti,ab,kw OR capramol:ti,ab,kw OR caproamin:ti,ab,kw OR caprogel:ti,ab,kw) OR ('e aminocaproic acid':ti,ab,kw OR eaca:ti,ab,kw OR 'epsilon amino caproic acid':ti,ab,kw) OR 'epsilon aminocaproic acid':ti,ab,kw OR (hemocaprol:ti,ab,kw OR ipsilon:ti,ab,kw) OR 'aprotinin'/de OR (antagosan:ti,ab,kw OR antilysin:ti,ab,kw OR antilysine:ti,ab,kw OR apronitin:ti,ab,kw OR apronitine:ti,ab,kw OR aprotinine:ti,ab,kw OR aprotonin:ti,ab,kw OR contrical:ti,ab,kw OR contrycal:ti,ab,kw OR contrykal:ti,ab,kw) OR (iniprol:ti,ab,kw OR 'kunitz inhibitor':ti,ab,kw OR 'kunitz trypsin inhibitor':ti,ab,kw) OR ('pancreatic antitrypsin':ti,ab,kw OR 'pancreatic secretory trypsin inhibitor':ti,ab,kw OR 'pancreatic trypsin inhibitor':ti,ab,kw OR trasilol:ti,ab,kw OR trasylol:ti,ab,kw OR 'tumor associated trypsin inhibitor':ti,ab,kw) OR ('4 aminomethylbenzoic acid':ti,ab,kw OR 'amino methylbenzoic acid':ti,ab,kw OR 'aminomethyl benzoic acid':ti,ab,kw OR 'aminomethylbenzoic acid':ti,ab,kw OR pamba:ti,ab,kw))

# **Table S3.** Excluded studies

| **Reason for exclusion** | **References** |
| --- | --- |
| Abstracts awaiting classification | Roos Y, Rinkel GJE, Vermeij FH, et al. Antifibrinolytic treatment in subarachnoid haemorrhage: study of tranexamic acid after aneurysmal rupture (STAR study). Cerebrovascular diseases.1998;8:20 |
| Abstracts awaiting classification | Bosch J, Nunez S, Vilalta J, et al. Risk factors, aetiology and therapy of subarachnoid haemorrhages. A retrospective study of 201 cases. Revista de Neurologia.1987;15:77:211-218 |
| Abstracts awaiting classification | Vermeulen M, Van Crevel H, Van Gijn J. Antifibrinolytic therapy in subarachnoid hemorrhage. Nederlands Tijdschrift voor Geneeskunde. 1986;130:8:345-348 |
| Abstracts awaiting classification | Kaste M, Ramsay M. Antifibrinolytic treatment of patients with subarachnoid hemorrhage. Acta Neurochirurgica.1979:51:1:131 |
| Background article | Post R, Germans MR, Vandertop WP, et al. Tranexamic acid for subarachnoid haemorrhage - Authors' reply. Lancet. 2021;3:398:25. |
| Background article | Nina P, Schisano G.Antifibrinolytic therapy. Neurosurgery. 2011;68:6:E1776-E1776 |
| Background article | N.A.Sunde. Antifibrinolytic therapy in subarachnoid hemorrhage.Ugeskrift for laeger. 2010;172:19:1431-1431 |
| Background article | Diringer, M N.Emerging trend: new therapy for ruptured intracranial aneurysms. Critical Care Medicine.2009;37:2:432-440 |
| Background article | Iplikcioglu AC, Bek S. The management of ruptured intracranial aneurysms. Journal of neurosurgery. 2003;98:5:1147 |
| Background article | Schisano G,Nina P. Experiences in management of ruptured aneurysms. Journal of neurosurgery 1975;87:3:486-487 |
| Background article | Neil-Dwyer G, Sharp MM, Sharr MM. Ruptured intracranial aneurysms. Journal of neurology. 1983;46:9:366 |
| Background article | Karis R.Antifibrinolytic therapy in subarachnoid hemorrhage. Neurology.1981;31:11:1498-1500 |
| Background article | Lindsay KW, Volo G, Teasdale GM. Antifibrinolytic therapy in subarachnoid hemorrhage. Journal of neurosurgery.1981;55:1:155-156 |
| Background article | Check W. Emerging trend: new therapy for ruptured intracranial aneurysms. JAMA.1978;240:20:2135-2136 |
| Background article | Gunasekera WS. The management of ruptured intracranial aneurysms. The Ceylon medical journal.1978;23:2:45-52 |
| Background article | Van Rossum J, Wintzen AR, Maurice-Williams RS. Antifibrinolytic agents for ruptured intracranial aneurysms. British medical journal.1978;2:6136:831-832 |
| Background article | Uttley D.Experiences in management of ruptured aneurysms. Journal of neurosurgical sciences.1975;19:1:65-68 |
| Other reasons for exclusion | Fodstad H.Tranexamic acid as therapeutic agent in aneurysmal subarachnoid hemorrhage.Neurochirurgia.1981;24:333 |
| Other reasons for exclusion | Kaste M, Ramsay M. Effect of tranexamic acid on fatal rebleeds after subarachnoid haemorrhage Double blind study. Acta neurologica scandinavica supplementum.1978;57:67:254 |
| Wrong design | Germans MR, Tjerkstra MA, Post R, et al. Impact of time to start of tranexamic acid treatment on rebleed risk and outcome in aneurysmal subarachnoid hemorrhage. Eur Stroke J. 2024;9658-666. |
| Wrong design | Germans MR, Tjerkstra M, Post R, et al. TRANEXAMIC ACID AFTER ANEURYSMAL SUBARACHNOID HEMORRHAGE: POST-HOC ANALYSIS OF A RANDOMIZED CONTROLLED TRIAL. European stroke journal.2022;7:1:63-63 |
| Wrong design | Tjerkstra MA, Post R, Germans MR, et al.Tranexamic Acid After Aneurysmal Subarachnoid Hemorrhage: post Hoc Analysis of the ULTRA Trial.Neurology.2022;99:23:e2605-e2614 |
| Wrong design | Rabinstein AA. Antifibrinolytics in subarachnoid haemorrhage. Lancet. 2021;397:10269:74-75 |
| Wrong design | Ultra-early tranexamic acid after subarachnoid hemorrhage.NTR3272. 2019;3 |
| Wrong design | Watanabe H, Ito M, Chigasaki H, et al. Antifibrinolytic therapy in ruptured intracranial aneurysm through repeated monitoring of fibrinolytic activity of blood Neurologia Medico-Chirurgica.1976;91-96 |
| Wrong design | Tovi D.Preoperative management of patients with aneurysmal subarachnoid hemorrhage.Journal of Neurosurgical Sciences. 1975;19:1:59-64 |
| Wrong design | Tovi D, Nilsson IM, Thulin CA. Fibrinolysis and subarachnoid haemorrhage. Inhibitory effect of tranexamic acid. A clinical study.Acta neurologica Scandinavica.1972;48:4:393-402 |
| Wrong design | Eastin TR, Snipes CD, Seupaul RA. Are antifibrinolytic agents effective in the treatment of aneurysmal subarachnoid hemorrhage? Annals of Emergency Medicine.2014;64:6:658-659 |
| Wrong design | Kassell NF, Torner JC, Adams Jr.HP.Antifibrinolytic therapy in the acute period following aneurysmal subarachnoid hemorrhage. Preliminary observations from the Cooperative Aneurysm Study.Journal of Neurosurgery.1984;61:2:225-230 |
| Wrong intervention | Adeoye O, Ferioli S, Meganathan, et al. Antifibrinolytic use after subarachnoid hemorrhage in the united states Neurocritical Care. 2011;15:1:S89-S89 |
| Wrong intervention | Larsen CC, Eskesen V, Hauerberg J, et al. Considerable delay in diagnosis and acute management of subarachnoid haemorrhage.Danish Medical Bulletin.2010;57:4 |
| Wrong intervention | Sorimachi T.Methods of antifibrinolytics administration and their side effects.Neurosurgery.2006;58:5:E1003 |
| Wrong intervention | Gutknecht JL, Irthum B, Cavaroc G, et al. [Evaluation of the treatment of aneurysmal meningeal hemorrhage with antifibrinolytic agents, calcium inhibitors and maintenance of effective blood volume]. Agressologie: revue internationale de physio-biologie et de pharmacologie appliquees aux effets de l'agression.1990;31:6:340-343 |
| Wrong intervention | Spallone A, Pastore FS, Rizzo A, et al. Low-dose tranexamic acid combined with aprotinin in the pre-operative management of ruptured intracranial aneurysms. Neurochirurgia.1987;30:6:172-176 |
| Wrong intervention | Chowdhary UM. Comparative clinical trial of epsilon-amino-caproic acid (EACA) and tranexamic acid (TEA) in prevention of early recurrence of sub-arachnoid haemorrhage (SAH) Acta neurochirurgica.1980;55:151-151 |
| Wrong intervention | Mullan S, Hanlon K, Brown F. Management of 136 consecutive supratentorial berry aneurysms. Journal of neurosurgery.1978;49:6:794-804 |
| Wrong intervention | Nibbelink DW, Torner JC, Henderson WG. Intracranial aneurysms and subarachnoid hemorrhage. A cooperative study. Antifibrinolytic therapy in recent onset subarachnoid hemorrhage.Stroke.1975;6:6:622-629 |
| Wrong intervention | Schisano G.Antifibrinolytics in the treatment of subarachnoid hemorrhages due to ruptured aneurysms. Journal of Neurosurgical Sciences. 1975;19:1:79-80 |
| Wrong population | Mozafari J. Evaluation of therapeutic effect of Tranexamic Acidinfusion during neurosurgery.IRCT2016031027003N1.2016 |
| Wrong population | A Spallone, FS Pastore, A Rizzo, et al. Risk facto  rs, aetiology and therapy of subarachnoid haemorrhages. A retrospective study of 201 cases. Neurochirurgia 1987;30:172-176 |
| Wrong population | Gutknecht JL, Irthum B, Cavaroc G, et al. Evaluation of the treatment of aneurysmal meningeal hemorrhage with antifibrinolytic agents, calcium inhibitors and maintenance of effective blood volume. Agressologie. 1990 Jun;31:340-343. |
| Protocols without results | Peter YM Woo. Tranexamic Acid for Spontaneous Acute Cerebral Hemorrhage Trial. NCT03044184.2018;5 |

# **Table S4**: Characteristics of included NRSI

| **Reference** | **Country** | Analysis method | **Number of patients; total (intervention/control)** | **Intervention （drug dosage, route, duration）** | Time from symptom onset to treatment | **Ischemia prophylaxis** |
| --- | --- | --- | --- | --- | --- | --- |
| Gibbs 1971 | England | Univariate analysis | 47 (25/22) | TXA 1 g/8 h, orally, 3 weeks | Unclear | Unclear |
| Garijo 1980 | Spain | Univariate analysis | 23 (11/12) | TXA 0.5 g/6 h, intravenously, 6 days, followed by 0.5 g/6 h, orally, days 7–21 | Unclear | Unclear |
| Gelmers 1980 | The Netherlands | Univariate analysis | 57 (31/26) | TXA 1 g/6 h, intravenously, until surgery or discharge | Within 72 h | Unclear |
| Beguelin 1983 | Switzerland | Univariate analysis | 42 (13/29) | TXA 6 g/day, administration method unclear, 3 weeks | Within 2 to 3 days | Unclear |
| Chowdhary 1986 | Saudi Arabia | Univariate analysis | 129 (65/64) | TXA 1 g/4 h, intravenously or orally, until the surgery or discharge | Unclear | Unclear |
| Pinna 1988 | Italy | Univariate analysis | 350 (260/90) | TXA 1 g/4 h, intravenously, 4 weeks | Within 7 days | Unclear |
| Wijdicks 1989 | England | Univariate analysis | 360 (119/241) | TXA, 1 g/4 h, intravenously, 96 h | Within 72 | Hypervolemia to prevent DCI |
| Post  2019 | The Netherlands | Multivariate logistic regression | 509 (119/390) | TXA 1 g bolus+1 g/8 h, intravenously, 1.5 day | Within 24 h | Nimodipine, hypertensive therapy, and normovolemia |
| Rynkowski 2023 | Brazil | Multivariate logistic regression | 230 (129/101) | TXA 1 g bolus+1 g/6 h, intravenously, 3 days | Within 72 h | Unclear |

All comparators in the NRSI were standard therapy.
DCI, delayed cerebral ischemia; TXA, tranexamic acid; NRSI, non-randomized studies of interventions

# **Figure S1.** Risk of bias in the included studies evaluating the neurological outcome.

(a) Randomized controlled trials.


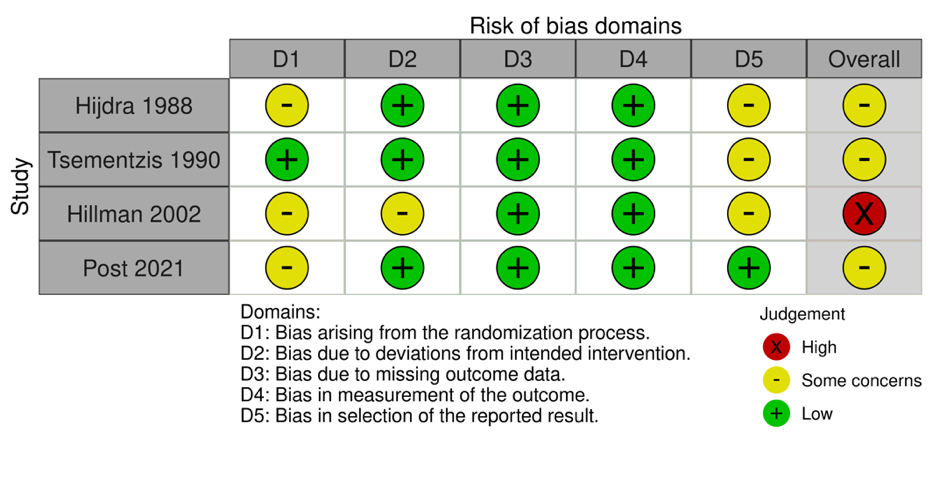


(b) Non-randomized studies of interventions.

# **Figure S2.** Risk of bias in the included studies evaluating the rebleeding.

(a) Randomized controlled trials.


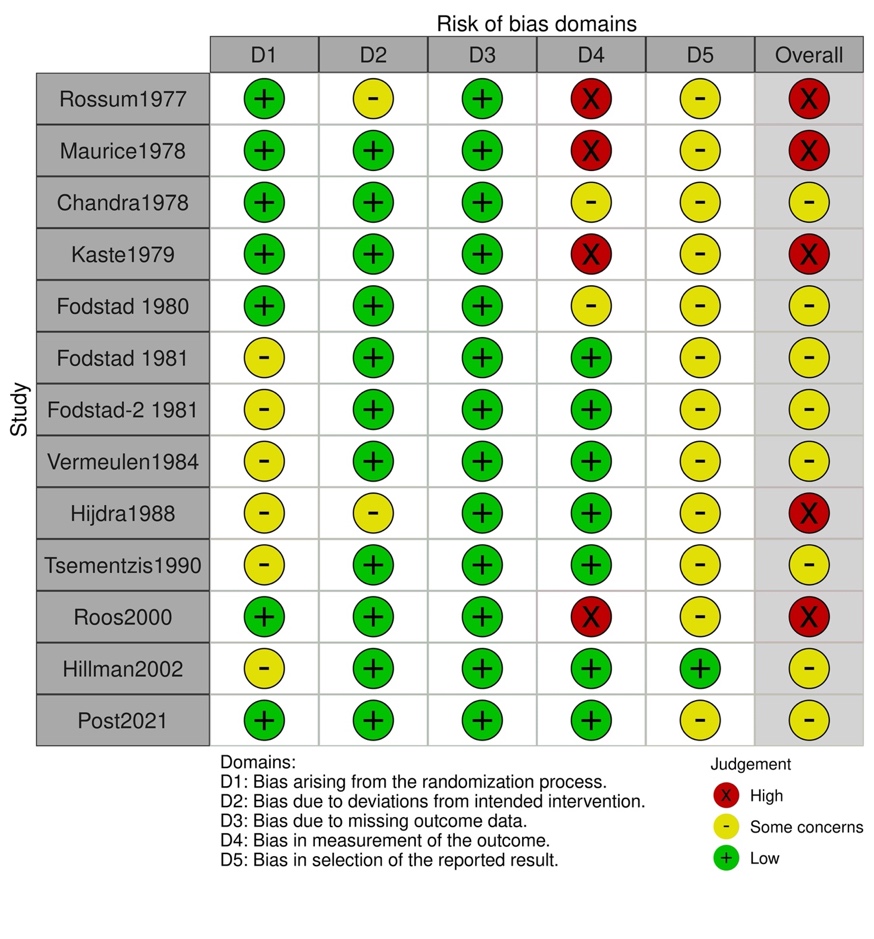


(b) Non-randomized studies of interventions.

# **Figure S3.** Risk of bias in the included studies evaluating thromboembolism.

(a) Randomized controlled trials.

(b) Non-randomized studies of interventions

# **Figure S4.** Risk of bias in the included studies evaluating the DCI.

(a) Randomized controlled trials.

(b) Non-randomized studies of interventions.

# **Figure S5.** Risk of bias in the included studies evaluating the hydrocephalus.

(a) Randomized controlled trials.

(b) Non-randomized studies of interventions.

# **Figure S6.** Risk-benefit diagram plotting for rebleeding and hydrocephalus.

Among studies in which TXA administration was associated with reduced hemorrhage volume, some reported a decreased risk of hydrocephalus, while others observed an increased risk.

# **Figure S7.** Funnel plot assessing publication bias for the mortality.


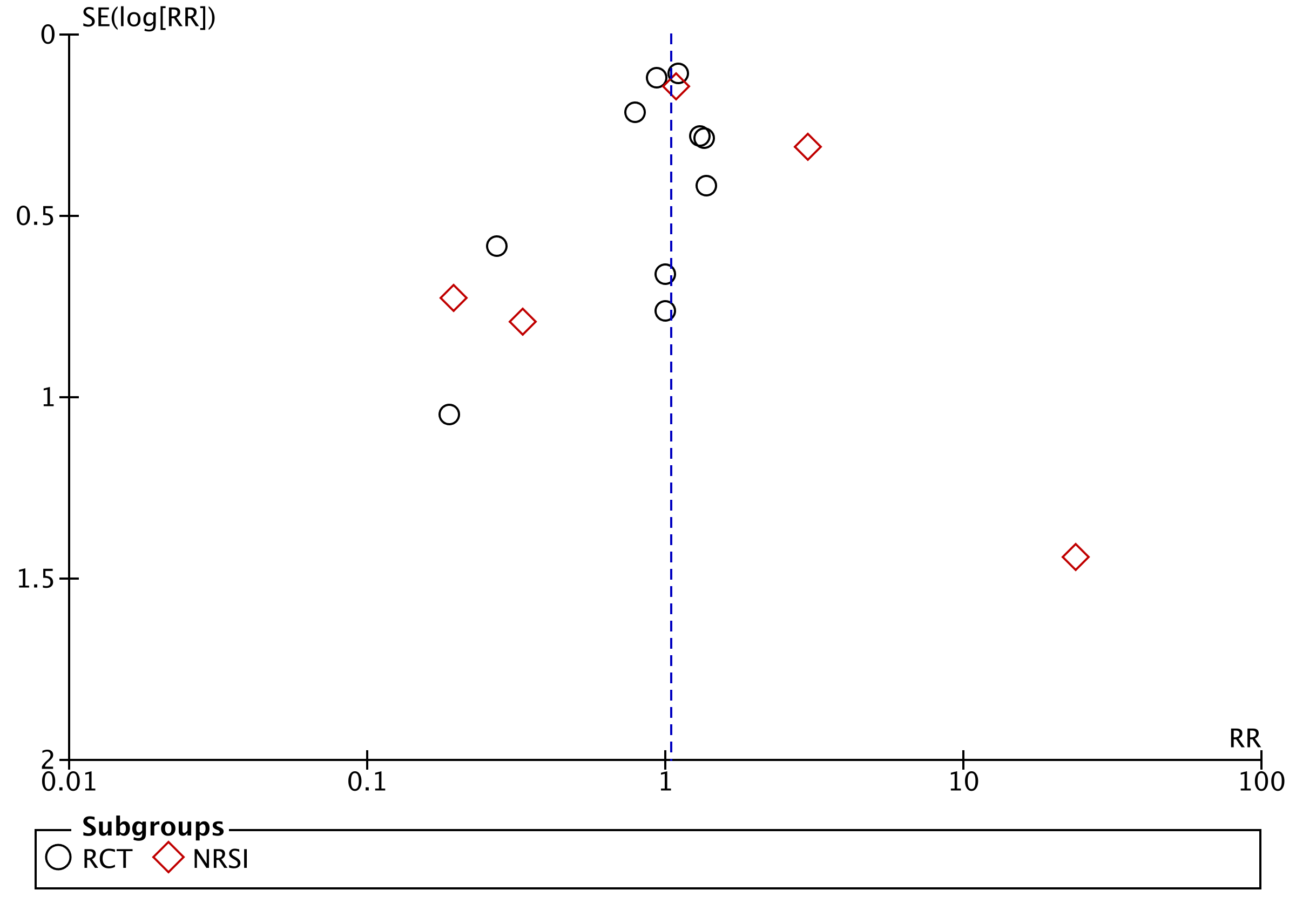


# **Figure S8.** Funnel plot assessing publication bias for the neurological outcome.


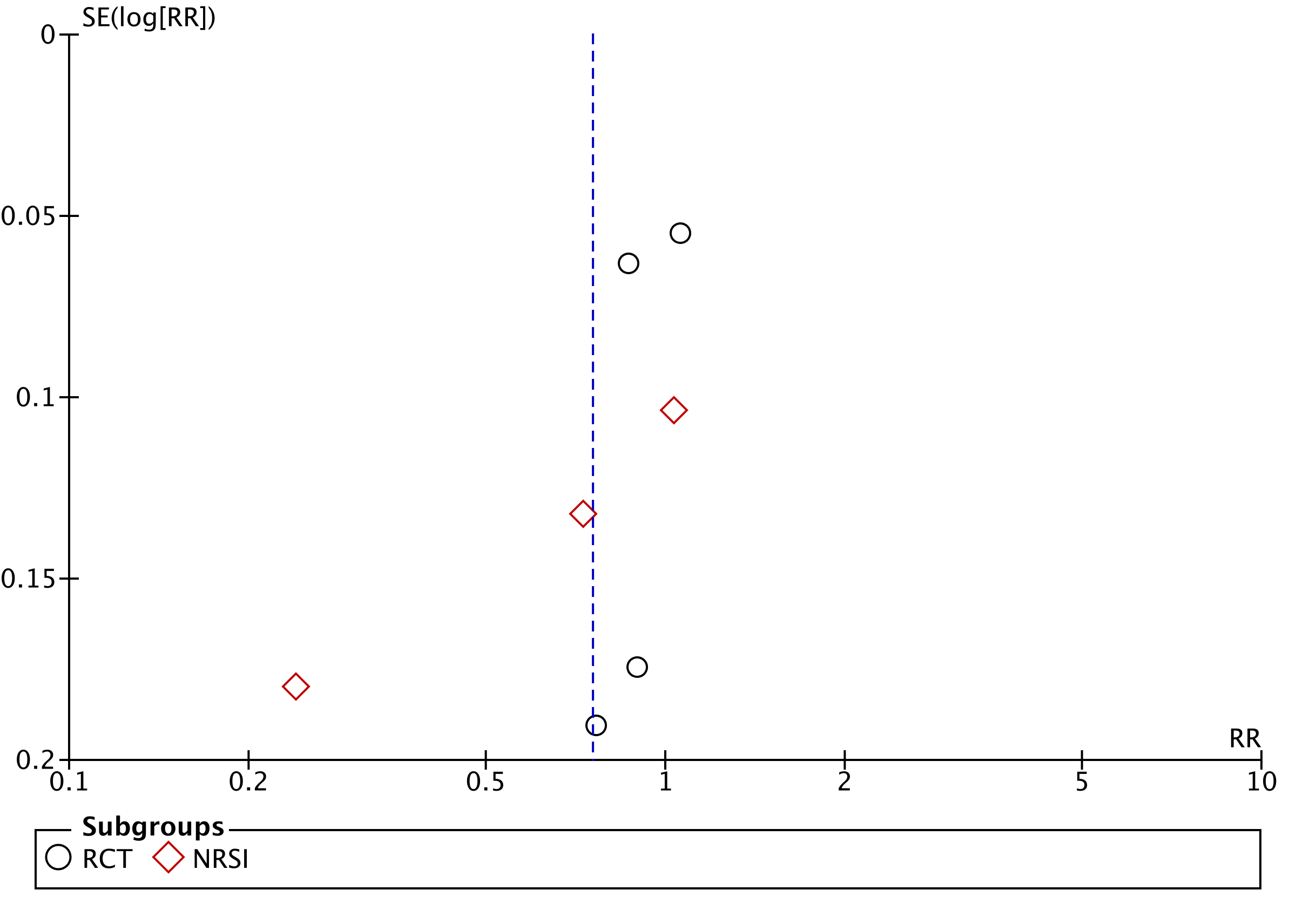


# **Figure S9.** Funnel plot assessing publication bias for the rebleeding.


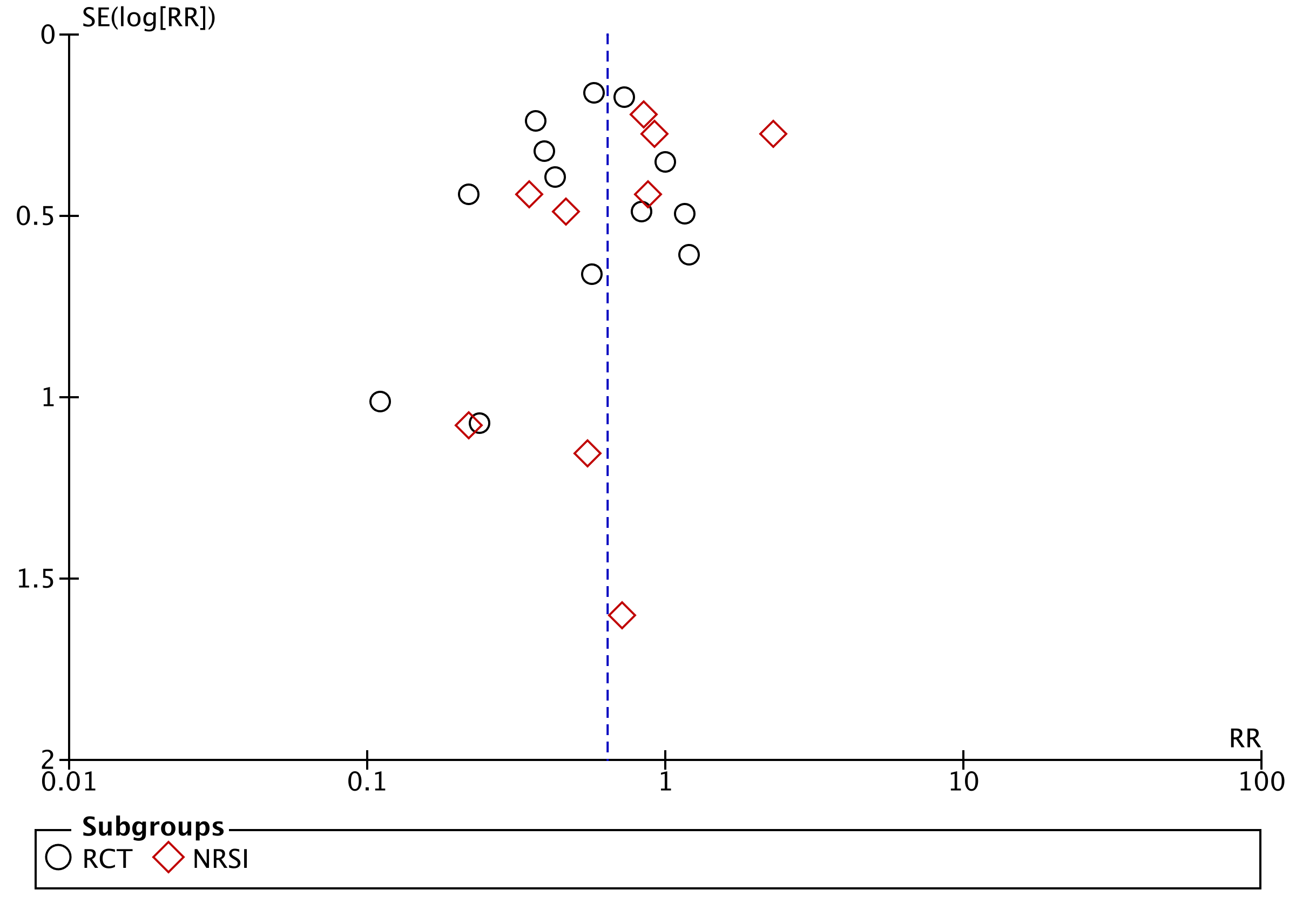


# **Figure S10.** Funnel plot assessing publication bias for the thromboembolism.

**
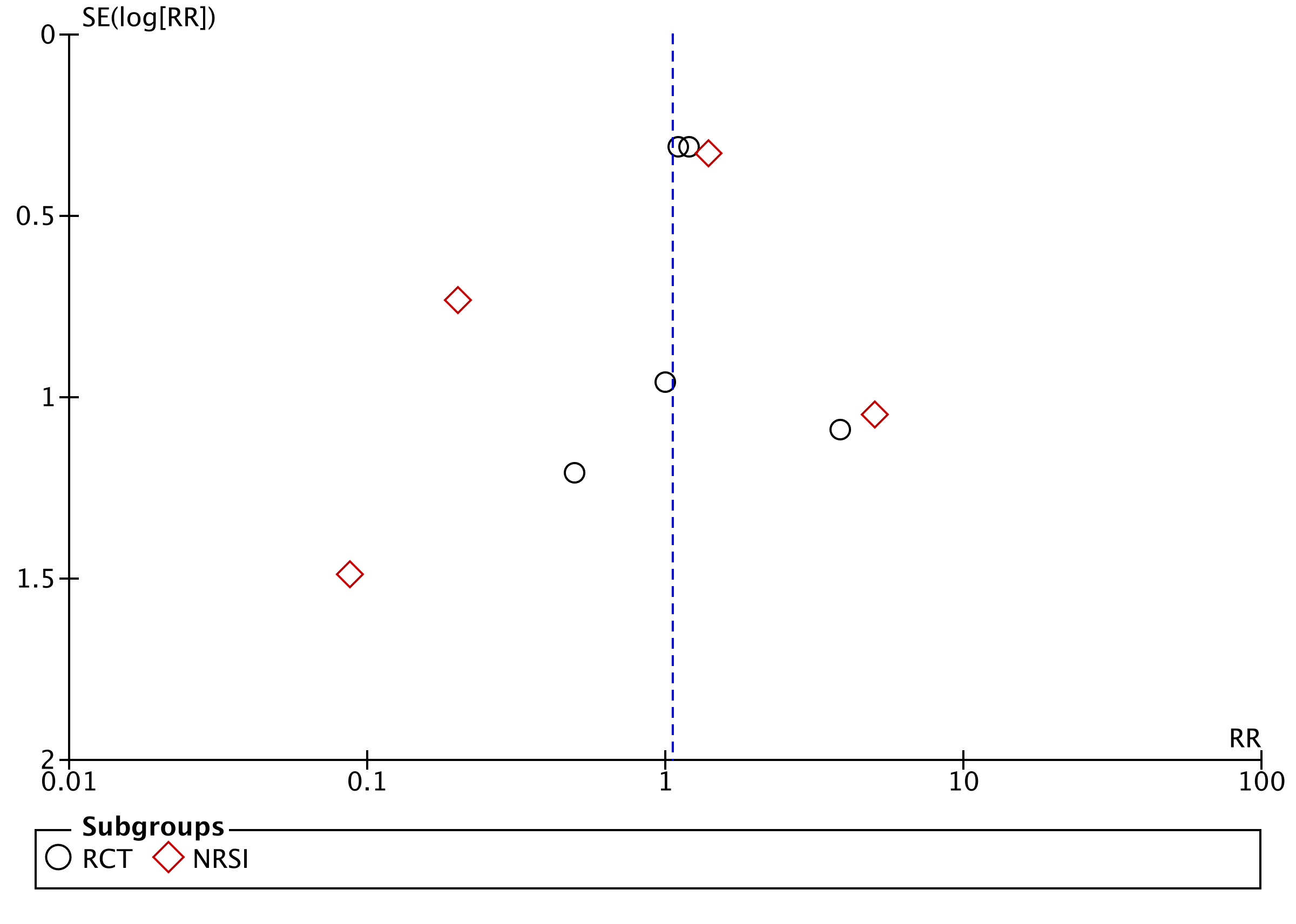
**

# **Figure S11.** Funnel plot assessing publication bias for DCI.


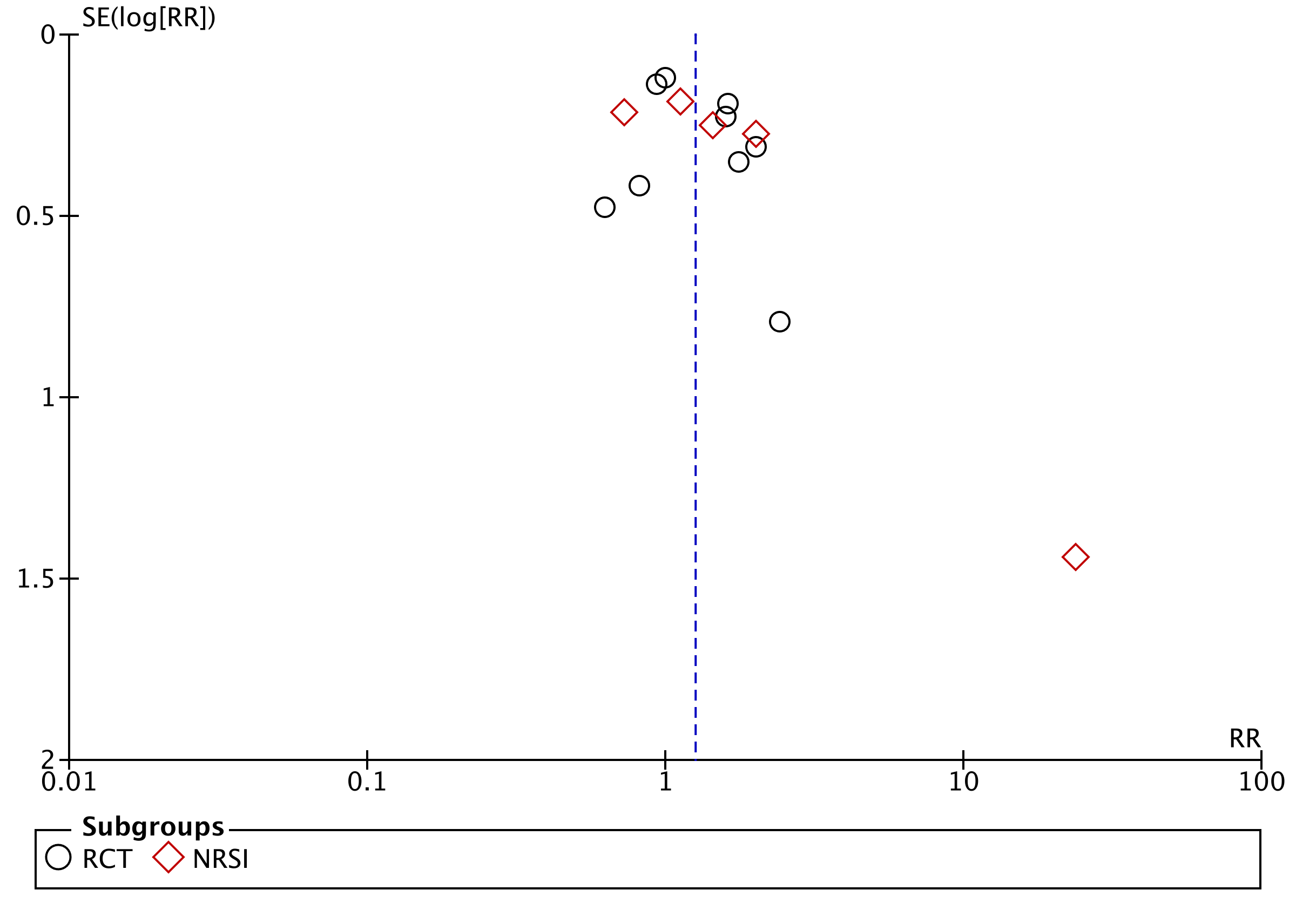


# **Figure S12.** Funnel plot assessing publication bias for hydrocephalus.


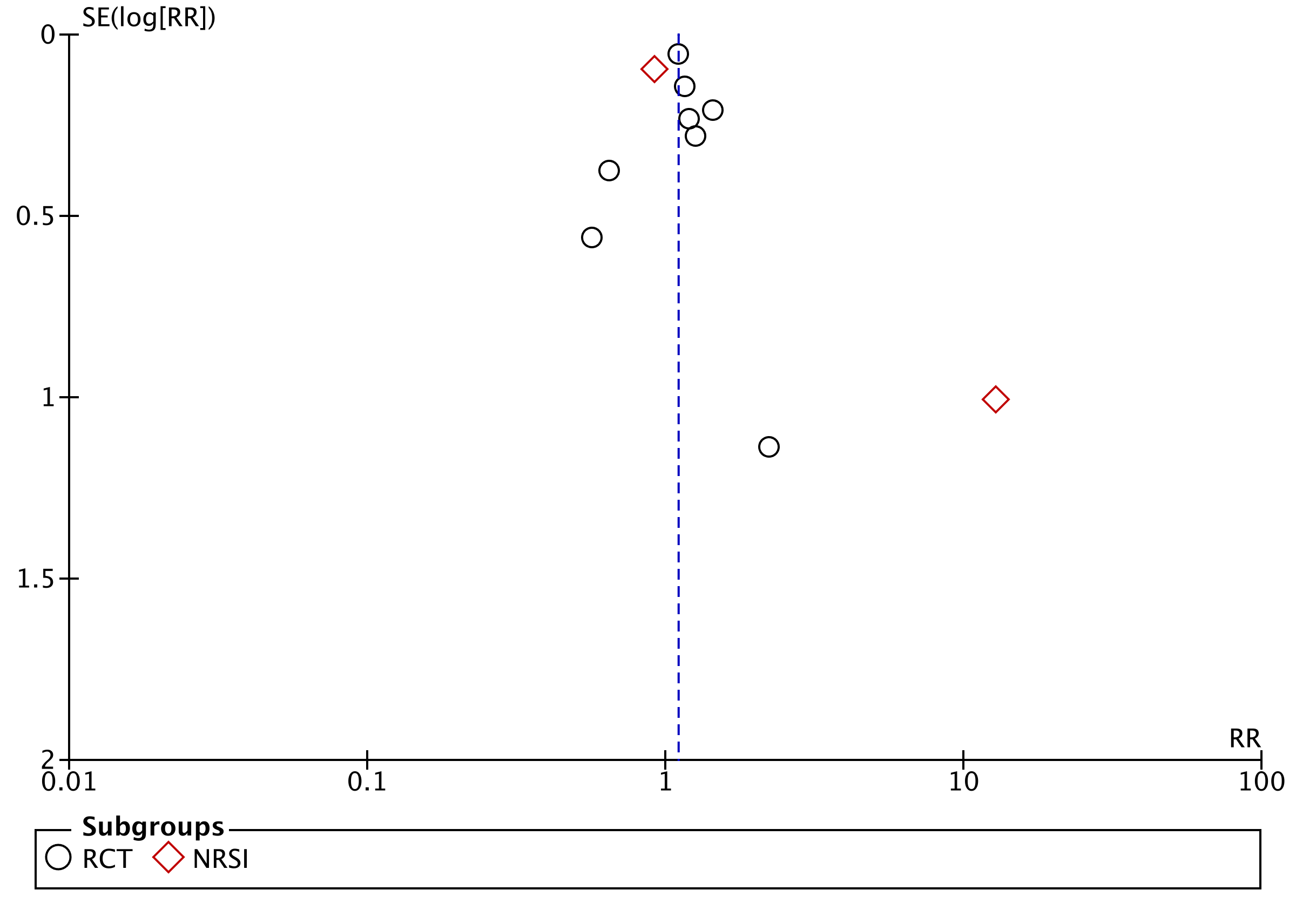


# **Figure S13.** Forest plot of subgroup analysis in mortality.

CI, confidence interval; df, degrees of freedom; MH, Mantel–Haenszel; SD, standard deviation; TXA, tranexamic acid.

# **Figure S14.** Forest plot of sensitivity analysis in mortality.


CI, confidence interval; df, degrees of freedom; MH, Mantel–Haenszel; SD, standard deviation.
